# O012: Can DAV132, a medical device targeting an adsorbent to the late ileum, decrease significantly the impact of antibiotics on the fecal microbiota?

**DOI:** 10.1186/2047-2994-2-S1-O12

**Published:** 2013-06-20

**Authors:** N Grall, E Chachaty, S Sayah-Jeanne, J de Gunzburg, A Andremont

**Affiliations:** 1 Lab. de Bacteriologie Hop. Bichat Claude-Bernard, Paris, France; 2 Institut Gustave-Roussy, Villejuif , France; 3 Da Volterra, Paris, France

## Introduction

During antibiotic treatments a fraction of the dose impacts the intestinal microbiota, promoting spread of resistant bacteria (RB). Antibiotics can be inactivated by adsorption. DAV132 is an oral medical device to deliver an adsorbent in the late ileum. We investigated if DAV132 could reduce fecal antibiotics and RB excreted.

## Methods

Three models were used. First, to explore the relationship between antibiotic exposure and excretion of RB, piglets just received 15 or 1.5 mg/Kg/d of oral ciprofloxacin or placebo X 5 days. We then compared between groups fecal ciprofloxacin concentrations and the amount of RB excreted. Second, to explore colonic adsorption of antibiotics by DAV132 in the colon, dogs received 10.7 mg/kg/d of IV levofloxacin X 5 days together with 0.3 or 0.6 g/kg/d or placebo of oral DAV132. We then compared between groups fecal and blood levofloxacin concentrations. Last, to explore if DAV132 could restore antibiotic-associated disruption of colonization resistance (CR), mice received 300 mg/kg/d of sc cefotaxime or placebo X 3 days, together with 50 mg/d or placebo of oral DAV131 (DAV132 adapted to the mice), followed by gastric challenge with 10^6^ CFU of a *K. pneumoniae* resistant to third generation cephalosporin (C3GR-Kp). We then compared between groups fecal cefotaxime and C3GR-Kp concentrations.

## Results

In piglets, counts of RB excreted were 9.2, 8.8 and 6.2 log10 CFU in animals receiving respectively the 15, the 1.5 mg/kg/d and the placebo regimen respectively (p<0.001). In dogs, reduction of fecal levofloxacin reached 71 and 82% when 0.3 or 0.6 g/kg/d of DAV132 was given. Blood PK of levofloxacin was not modified significantly. In mice, all antibiotic disappeared from the pellets when DAV131 was given to the animals together with cefotaxime and a significant part of RC by C3GR-Kp was restored.

## Conclusion

Oral DAV132 might reduce exposure of the intestinal flora by antibiotics which could be associated with decrease in fecal excretion of RB without affecting blood PK. There appeared to be a relationship between the dose of DAV132 administered and the effect observed. The possible clinical use of DAV132 is under investigation.

## Disclosure of interest

N. Grall: None declared, E. Chachaty Consultant for DA VOLTERRA, S. Sayah-Jeanne Employee of DA VOLTERRA, J. de Gunzburg Shareholder of DA VOLTERRA, A. Andremont Consultant for DA VOLTERRA.

